# Neem Essential Oil as an Antifungal Agent against *Phyllosticta citricarpa*

**DOI:** 10.1155/2024/6251407

**Published:** 2024-07-19

**Authors:** Joyce Maria Schuch, Carolina Rosai Mendes, Guilherme Lopes Cardoso, Carlos André da Veiga Lima Rosa Costamilan, Paulo Renato Matos Lopes, Renato Nallin Montagnolli, Guilherme Dilarri, Ederio Dino Bidoia

**Affiliations:** ^1^ Department of General and Applied Biology Sao Paulo State University (UNESP), Avenida 24-A 1515 Postal Code: 13506-900, Rio Claro, SP, Brazil; ^2^ Department of Fisheries Engineering and Biological Sciences Santa Catarina State University (UDESC), Rua Coronel Fernandes Martins 270 Postal Code: 88790-000, Laguna, SC, Brazil; ^3^ College of Technology and Agricultural Sciences Sao Paulo State University (UNESP), SP-294 Km 651 Postal Code: 17900-000, Dracena, SP, Brazil; ^4^ Department of Natural Sciences Mathematics and Education Federal University of Sao Carlos (UFSCar), SP-330 Km 174 Postal Code: 13600-970, Araras, SP, Brazil; ^5^ Multicentric Graduate Program in Biochemistry and Molecular Biology (PMBqBM) Santa Catarina State University (UDESC), Avenida Luiz de Camões 2090 Postal Code: 88520-000, Lages, SC, Brazil

## Abstract

The fungus *Phyllosticta citricarpa* is a quarantine phytopathogen responsible for causing citrus black spot (CBS) disease. To export fruits to CBS-free countries, they must undergo a sanitation process to ensure disease control. In this study, neem essential oil (NEO) was tested against *P. citricarpa* for the first time as an alternative sanitizer. *In vitro* experiments were conducted to determine the inhibition concentration of NEO for *P. citricarpa*, and the mode of action of the essential oil was evaluated. *In vivo* assays were performed to simulate the sanitization process used in packinghouses. NEO was characterized by GC-MS/MS. The results revealed that NEO at 100 *μ*L·mL^−1^ exhibited a similar inhibitory effect as copper oxychloride, suppressing 89.68 ± 1.14% of fungal mycelium growth. Fluorescence microscopy experiments demonstrated that NEO functions by disrupting the cytoplasmic membrane of fungal hyphae, leading to their death within 30 minutes of contact with NEO. GC-MS/MS characterization revealed a high presence of phenolic compounds, which serve as the primary antifungal agents responsible for the action against fungal hyphae. *In vivo* assays showed that NEO at 100 *μ*L·mL^−1^ also reduced microorganisms (CFU mL^−1^) by 93.00 ± 3.88% compared to the negative control. Overall, the results demonstrate that NEO can effectively serve as an alternative sanitizer against *P. citricarpa* in citrus packinghouses. Our findings allow future studies to explore the use of NEO for sanitizing other fruits and combating different phytopathogens to broaden its potential application in fruit sanitation for export.

## 1. Introduction

The orange fresh fruit, a quintessential agricultural product, has annual export revenue of approximately 2 billion US dollars worldwide, rendering it a cornerstone commodity for major producer countries like the USA, Brazil, and China, boasting remarkable profitability [1, 2]. Within the USA, fresh citrus fruits, including oranges, constitute one-fifth of all fresh fruit consumption, playing a pivotal role in the American diet [3]. Not only are citrus fruits esteemed for their flavor but they also harbor significant antioxidant properties, brimming with phytochemicals and vitamins such as Vitamin C, which impart its many health benefits [4, 5]. However, this agricultural treasure is not without its challenges; natural phytopathogens inhabit citrus fruits, compromising their quality for consumption. Citrus is susceptible to quarantine diseases, imposing restrictions on their exportation and distribution to consumers. Among these quarantine diseases, citrus black spot (CBS) emerges as one of the most formidable adversaries, inflicting substantial losses upon producers [6, 7]. *Phyllosticta citricarpa*, the causative agent of CBS, ravages economically significant citrus species, precipitating fruit depreciation and orchard yield diminishment [8, 9]. The lifecycle of *P. citricarpa* within citrus orchards is complex, with contamination of leaves and fruits being commonplace [10, 11]. Notably, *P. citricarpa* spores can adhere to citrus fruit surfaces, with disease symptoms manifesting after approximately 40 days, although the majority tend to emerge closer between 110 to 200 days [11–13]. Symptoms may only become apparent as the fruit ripens or during postharvest stages, facilitating disease dissemination to new regions and countries. Therefore, it is imperative that countries grappling with CBS ensure the thorough eradication of *P. citricarpa* propagules, including mycelia and spores, from the fruit surfaces earmarked for exportation.

The CBS has entrenched itself in the USA and Brazil [1, 14], both major citrus producing countries. European Union classified the CBS as A1 quarantine phytopathogen (Phytosanitary Regulations 2019/2072), having a high control for the citrus fresh fruits exported to their countries [15, 16]. Therefore, to control the CBS and avoid its dissemination to the free disease areas, it is applied fungicides based on copper to protect the fruits during the period of susceptibility [17]. Copper oxychloride at 90 g·L^−1^ of concentration is the mainly recommended copper-based formulation to control CBS [8, 18]. The Brazilian Ministry of Agriculture, Livestock, and Supply (MAPA) mandates thorough sanitation of citrus fruits at packinghouse facilities to ensure the eradication of any lingering phytopathogens on fruit surfaces [19]. Sanitizing agents utilized in this process must demonstrate efficacy against the targeted phytopathogen to guarantee the complete elimination of disease propagules [1, 19]. The current prevalent sanitation practice at Brazilian citrus packinghouses involves immersing fruits in a sodium hypochlorite solution at 200 ppm for a duration of 2 minutes [1]. So far, no study has been carried out to find a specific sanitizer for *P*. *citricarpa* that still has a residual effect and eliminates other microorganisms present on the citrus surface.

Essential oils have surfaced as a promising alternative amidst the emergence of novel organic sanitizers, particularly since their approval by the European Union Commission Regulation as suitable sanitizing agents for citrus fruits [1, 20, 21]. Essential oils are extracted from plant leaves, while vegetable oils are generally extracted from seeds. Despite originating from the same plant, essential oils may exhibit variations in composition owing to their extraction from different plant parts. A myriad of essential oils has been recognized for their potent antimicrobial properties on inhibiting microbial cells and biofilms [22]. Most notably, *Azadirachta indica* essential oil, commonly referred to as neem essential oil (NEO), has demonstrated bactericidal efficacy against an array of clinical pathogens, in addition to serving as a natural mosquito repellent for textiles [23–25]. Neem leaf extract has also high efficacy in limiting the growth of fungi of the genus *Rhizopus* [26]. Furthermore, studies have showcased the antifungal activity of NEO against postharvest pathogens such as *Penicillium expansum*, *Monilinia fructicola*, and *Trichothecium roseum* [27]. NEO harbors a diverse array of therapeutic properties attributed to alkaloid, flavonoid, saponin, and steroid metabolites [28, 29]. Active compounds within NEO are categorized into two principal groups: isoprenoids and nonisoprenoids. Isoprenoids encompass triterpenoids and diterpenoids, including limonoids, gedunin, protomeliasin, and azadiron. Nonisoprenoid compounds comprise sulfur, proteins, dihydrochalcone, carbohydrates, glycosides, and polyphenols [29]. Neem leaves additionally harbor nimbin, nimbandiol, 6-desacetylnimbine, nimbanene, nimbolide, n-hexacosanol, ascorbic acid, nimbiol amino acid, 17-hydroxyazadiradione, 7-desacetyl-7-benzoylgedunine, and 7-desacetyl-7-benzoylazadiradione [28]. Many of these organic chemical constituents within NEO exhibit potent bactericidal and fungicidal properties. Despite NEO's remarkable antimicrobial attributes, its efficacy against *P. citricarpa* has not been evaluated, nor has it been assessed as a sanitizer compound for citrus fruits.

Therefore, the aims of the present study were to evaluate the antifungal effect of NEO against *P*. *citricarpa* and its possible use as a sanitizer agent in citrus fruit packinghouses. This is a novel research study that has never been conducted before. *In vitro* assays were used to determine the inhibition concentration of NEO for *P*. *citricarpa*. The antifungal molecules present in NEO were identified through GC-MS/MS analysis, which allowed for a clear understanding of their mechanisms of action against the *P*. *citricarpa* hyphae. *In vivo* assays were carried out to simulate a commercial citrus packinghouse and compare the action of NEO with the copper oxychloride (the major agent in CBS control), bringing a novel organic sanitizing agent against CBS for the citrus fresh fruits market.

## 2. Materials and Methods

### 2.1. Essential Oil

The NEO sourced from Laszlo® (Sao Paulo, Brazil), licensed under N° 160 and a member of the aromatherapy trade council, was utilized. The NEO was cold pressed from *A*. *indica* leaves, exhibiting approximately 97% purity extracts.

### 2.2. Phytopathogen

This study used *P. citricarpa* (McAlpine) van der Aa (synonym: *Guignardia citricarpa* Kiely) (32758-ATCC), stored in silica spheres as outlined by Lange and Boyd [30]. This strain is from the collection of the Laboratory of Multidisciplinary Sciences, Sao Paulo State University (UNESP), Rio Claro campus, Brazil. Cultivation of the fungus occurred on both liquid and solid YPD medium (10 g·L^−1^ of yeast extract, 20 g·L^−1^ of bacteriological peptone, 20 g·L^−1^ of dextrose, and 15 g·L^−1^ of agar for solid medium) over a 21-day period at 28 ± 1°C. All reagents for cellular growth were acquired from Himedia Laboratories Ltd. (Mumbai, India).

### 2.3. Antifungal Sensitivity Assays

The antifungal sensitivity assays to NEO were carried out by the growth of *P. citricarpa* in YPD agar plate, according to the methodology of CLSI AST guidelines [31]. A 10 mm radial fungal mycelium was placed at the center of an YPD agar medium plate. Firstly, the medium plates received different concentrations of the NEO before the *P. citricarpa* inoculation, where the essential oil concentrations in the medium were 40, 60, 80, and 100 *μ*L·mL^−1^. The NEO was diluted in 1% dimethylsulfoxide (DMSO) from Sigma-Aldrich (Darmstadt, Germany); therefore, for the negative control (NC), 100 *μ*L of autoclaved deionized water with 1% DMSO was used. The positive control (PC) used in the experiments was a commercial copper formulation called Difere®, manufactured by Oxiquímica Agrociência Ltda. (Jaboticabal, Brazil). It contains 350 g·L^−1^ of metallic copper and 588 g·L^−1^ of copper oxychloride. This solution is commonly used as a fungicide in citrus farming. The concentration of Difere® used as PC in the present work was 226.44 g·L^−1^ (90 g·L^−1^ of copper oxychloride in the final solution). The area of fungal growth after 21 days at 28 ± 1°C was measured using ImageJ software (open source), and the graphs were plotted using Origin version 8 (OriginLab Corporation, USA). All antifungal sensitivity assays comprised three replicas and were conducted thrice. Data validation was performed using the standard deviation (*SD*)

(1) $$\begin{array}{c} \text{SD}=\sqrt{\frac{1}{N-1}\sum_{i=1}^{N}{\left(\frac{\text{Qie}-\text{Qic}}{\text{Qie}}\right)}^{2},} \end{array}$$

in which Qie and Qic are experimental and calculated data and *N* is the number of measurements carried out.

### 2.4. Fluorescence Microscopy Assays

The membrane integrity of *P. citricarpa* was evaluated using the Live/Dead-Cell Viability Kit from Thermo Fisher (Waltham, USA). Prior to assessment, fungal mycelium was cultured in YPD broth at 28 ± 1°C with agitation at 150 rpm for 10 days. Then, the mycelium was separated by centrifugation at 4000 × *gForce* and remained in contact for 30 min with NEO at the optimal concentration for inhibiting *P. citricarpa*, as determined in the antifungal sensitivity assays. SYTO-9 and propidium iodide (PI) dyes, at a concentration of 50 *μ*M each, were employed to stain live (green) and dead (red) fungal hyphae, which were then observed under a fluorescence microscope. Fungal mycelium was immobilized on agarose-coated slides and examined using an Opticam O600R fluorescence microscope (Doral, USA), outfitted with a monochromatic Opticam 12.3 MP camera (Doral, USA). Image processing was conducted using OPTHD, Opticam software (Doral, USA). These assays were performed in triplicate, with a minimum of 50 hyphae visualized per analysis.

### 2.5. Characterization of NEO by Gas Chromatography Tandem Mass Spectrometry (GC-MS/MS)

GC-MS/MS analysis was employed to characterize the antifungal compounds present in NEO. The analysis was conducted using a Shimadzu GC-MS model QP-2010 Ultra gas chromatograph coupled with a flame ionization detector and mass spectrometer (GC-FID-MS) (Kyoto, Japan). A DB-5 fused silica column (300 mm × 0.25 mm × 1 *μ*m df) was utilized, with helium gas (1 mL·min^−1^) as the carrier at 89 kPa. NEO, extracted into ethyl acetate, was injected in a 10:1 split ratio mode. The initial injector temperature was maintained at 210°C for 2 minutes, followed by an increase to 280°C at a rate of 5°C/min. Electronic ionization at 70 eV and a scan rate of 0.5 scan/s were applied for sample analysis. MS1 level spectra processing parameters employing the Wavelet (ADAP) method were conducted according to Ni et al. [32]. All GC-MS data files (mzData) were processed using MZmine 2.53 software (open source), according to the parameters outlined by Elie et al. [33].

### 2.6. Sanitization of Fresh Fruits

The efficacy of NEO in eradicating *P. citricarpa* from citrus surfaces ensures the elimination of any *P. citricarpa* propagules, and thus sanitization assays mimicking packinghouse conditions were conducted. Fungal mycelium was cultured in YPD broth for 10 days at 28 ± 1°C under constant agitation of 150 rpm. Subsequently, the mycelium was separated via centrifugation at 4000 × *gForce* and resuspended in phosphate buffer (PBS) (137 mM NaCl, 2.7 mM KCl, 10 mM Na_2_HPO_4_, 1.8 mM KH_2_PO_4_). For sanitization, fresh *Citrus sinensis* (*L*.) cv. Pera fruits were washed with neutral soap (Wash Fruit Aruá®, Matão, Brazil) at a concentration of 33.5% to remove debris, followed by drying. The experiment comprised four independent replicates, with each treatment group consisting of 15 oranges, following the methodology by Zamuner et al. [21] with modifications. Initially, the fruits were inoculated with *P. citricarpa* by spraying 200 mL of a saline solution (0.87% of NaCl) with 10^6^ spores per mL of the fungus. The fruits were air-dried at room temperature (23 ± 1°C) for 240 minutes and then washed with NEO diluted in 1% DMSO at the concentration determined in the antifungal sensitivity assays. Autoclaved deionized water with 1% DMSO served as the negative control (NC), while Difere® at 226.44 g·L^−1^ (90 g·L^−1^ of copper oxychloride) served as the positive control (PC). The washing step entailed submerging the fruits in each respective treatment solution for 2 minutes, followed by air-drying (without heat) for 30 seconds to remove excess solution, simulating the immersion bath and drying process in a citrus packinghouse [1]. At last, the fruits were incubated for 40 days at 23 ± 1°C in a sterile chamber to prevent contamination with other microorganisms. After the incubation period, the fruits from each treatment were individually washed in PBS, and 100 *μ*L of the wash solution was spread on YPD agar plates and incubated for 21 days at 28 ± 1°C to facilitate *P. citricarpa* growth and assess complete elimination from the fruit surface. Microbial counts were conducted, and the data were subjected to nonparametric statistical analysis using Kruskal–Wallis (Dunn) with three degrees of freedom. All sanitization assays were performed in triplicate with four independent replicates. Data validation was performed using standard deviation (equation (1)) to ascertain experimental errors. Graphs were generated using Origin 8 software (OriginLab Corporation, USA), while statistical analyses were conducted using BioEstat 5.0 (open source).

## 3. Results and Discussion

**Figure 1:**
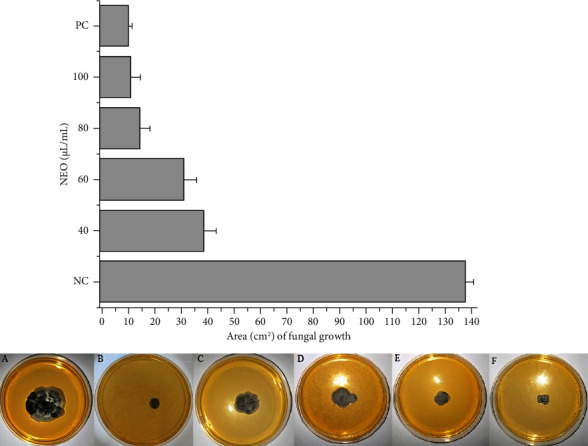
Comparative fungal growth of *P. citricarpa*, by area measured in the ImageJ software, after contact with different concentrations of NEO (40, 60, 80, and 100 *μ*L·mL^−1^). PC was 90 g·L^−1^ of copper oxychloride, and NC was deionized water with 1% of DMSO. The photos of fungal growth of *P. citricarpa* on YPD agar plates are showed below of the graph, where A is NC; B is PC; C is NEO at 40 *μ*L·mL^−1^ of concentration; D is NEO at 60 *μ*L·mL^−1^ of concentration; E is NEO at 80 *μ*L·mL^−1^ of concentration; F is NEO at 100 *μ*L·mL^−1^ of concentration. Bars represent the average values of fungal growth area (cm^2^); whiskers indicate the SD of the mean of three independent experiments used to verify experimental errors.

The antifungal sensitivity assays revealed promising results regarding the efficacy of NEO in inhibiting the growth of *P. citricarpa* (Figure 1). The fungal growth was notably affected by NEO, with the concentration of 100 *μ*L·mL^−1^ of the essential oil exhibiting a statistically equivalent inhibition to that of copper oxychloride (Figure 1). Furthermore, a concentration-dependent inhibition of *P. citricarpa* growth by NEO was observed, resulting in the formation of a dose-response curve. These findings underscore the specificity of NEO's activity, targeting the structural integrity of the fungal hyphae, rather than exerting a physicochemical influence akin to that of free chlorine, oxygen-containing reactive species (ROS), or pH [34]. The absence of a fixed dose-response inhibition, characterized by decay in inhibition relative to lower compound concentrations, is indicative of a physicochemical mode of action, as opposed to a targeted interaction with a specific cellular site inhibited by the antimicrobial agent. Khan et al. [35] similarly demonstrated via growth inhibition curves that neem oil operates via a specific mechanism against fungal hyphae, corroborating the outcomes of our study. Notably, Khan et al. [35] also reported favorable inhibition results using propyl disulfide from neem oil against *Lasiodiplodia theobromae* and *Neofusicoccum parvum*, with concentrations closely resembling those employed in our investigation.

Rodrigues et al. [36] similarly documented the inhibition of *Aspergillus carbonarius* using neem oil extracted from seeds, conducting experiments closely aligned with those in the present study. Rodrigues et al. [36] achieved approximately 95% inhibition of fungal growth with a concentration of 3 *μ*L·mL^−1^ of neem oil extracted from seeds. In comparison, our study employed 100 *μ*L·mL^−1^ to achieve a comparable inhibitory effect to that of copper oxychloride, resulting in 13.94 ± 1.33 cm^2^ of fungal growth area (equating to 89.68 ± 1.14% fungal growth inhibition). It is noteworthy that the comparatively lower results obtained in our study may stem from variances in neem oil composition and the species of fungus utilized, as opposed to those examined by Rodrigues et al. [36]. Furthermore, we attained a lower concentration of NEO relative to copper oxychloride at 90 g·L^−1^, while still achieving equivalent fungal inhibition. Additionally, Falsini et al. [37] demonstrated significant fungicidal efficacy with neem oil (extracted from both leaves and seeds) at a concentration of 1.5% when combined with a nanoformulation vehicle, thereby augmenting the inhibitory effect of neem oil. Despite this enhancement, the concentration applied and the hyphae inhibition observed closely paralleled our findings, substantiating the effectiveness of pure NEO tested against *P. citricarpa* in our study. The utilization of pure essential oil presents advantages in terms of cost-effectiveness for application in packinghouses or orchards, albeit requiring higher concentrations for efficacy. This highlights the importance of further experimentation and suggests the potential viability of utilizing pure essential oils, offering cost benefits for farmers and industries alike.

**Figure 2:**
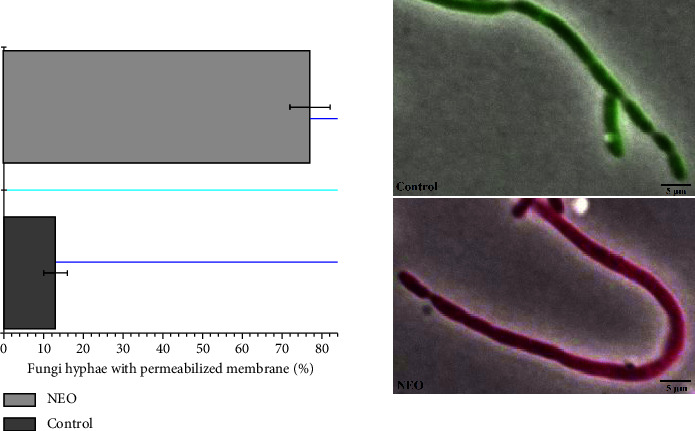
Percentage of *P. citricarpa* hyphae with the permeabilized cytoplasmic membrane. Hyphae with intact membranes are colored green, while the permeabilized membranes are colored red. The concentration of NEO was at 100 *μ*L·mL^−1^, and the control was a sterilized saline solution (NaCl 0.87%) with 1% of DMSO. The pictures showed were at the overlay of Tx Red/eGFP and phase contrast images, with a magnification of ×100. The experiment was performed thrice and at least 50 hyphae were evaluated per experiment (*n* > 150). Horizontal bars are the average percentage; whiskers are the average SD for verification of experimental errors.

To evaluate the action mechanisms of the NEO, fluorescence microscopy assays were carried out with the Live/Dead kit. In this method, the entry of propidium iodide into the cell, staining it red, indicates damage to the cytoplasmic membrane and subsequent cell death [38]. The results showed that the *P. citricarpa* hyphae were directly affected by the disruption of cytoplasmatic membrane after the contact with the NEO (Figure 2). NEO exhibited rapid action within the initial 30 minutes of contact, with approximately 76% of *P. citricarpa* hyphae affected by the oil (depicted as red-stained hyphae) (Figure 2).

The hyphae of the fungal mycelium exhibited a red stain following exposure to 100 *μ*L·mL^−1^ of NEO, indicating the targeted effect of NEO on the cytoplasmic membrane of *P. citricarpa*. The control group demonstrated intact cell cytoplasmic membranes (depicted as green hyphae), with no discernible effect of the vehicle (1% DMSO). Silva et al. [39] previously correlated the action mechanisms of NEO against fungi such as *Aspergillus flavus* and *Penicillium citrinum* with cytoplasmic membrane disruption. The findings of our study align with those of Silva et al. [39], further substantiating the effect of NEO on the cytoplasmic membrane. Essential oils, in general, exert their effects on the cytoplasmic membranes of fungi, ultimately leading to cellular cytoplasm lysis [40, 41]. Similar actions have been observed in bacteria such as *Klebsiella pneumoniae* [42]. This study marks the first observation of this action mechanism of NEO in *P. citricarpa*, underscoring the novelty of our results. Identifying the mechanism of action of antimicrobial compounds is crucial for analyzing potential mechanisms of resistance in microorganisms. Consequently, understanding the mechanism of action ensures the safe use of essential oils against phytopathogenic microorganisms. Based on our results, it can be inferred that NEO does not select or induce resistance in *P. citricarpa*, as developing resistance against NEO would necessitate multiple genetic mutations in the hyphae of *P. citricarpa* to alter the composition and structure of the cytoplasmic membrane [43, 44].

**Figure 3:**
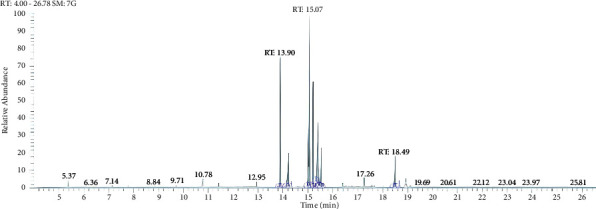
NEO spectral by GC-MS/MS.

The NEO characterization was done through GC-MS/MS. After identifying the different organic compounds found in NEO, it is possible to determine whether they have an active role in the antifungal action. Moreover, by performing GC-MS/MS analyses, the organic molecules can be identified, and it becomes possible to establish a correlation between the inhibitory mechanisms of action observed in fluorescence microscopy and the organic compounds present in the NEO. The mass spectral fragmentation patterns were compared with the National Institute of Standards and Technology Mass Spectral (NIST4-MS) database. The GC-MS/MS spectra displayed bands labeled according to retention time (RT) of different compounds identified by molecular weight (Figure 3). To identify the primary compounds, a table was compiled to provide further elucidation (Table 1).

**Figure 4:**
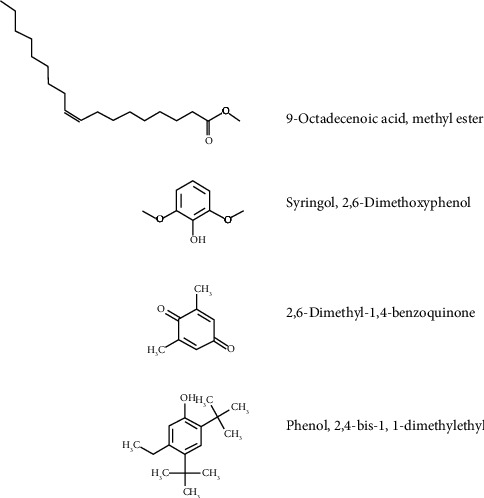
Molecular structure of compounds associated with the antifungal activity by NEO.

Four of the primary molecules identified in NEO are represented in Figure 4, all of which have been associated with antifungal activity in previous studies [45–49]. Among the organic molecules observed in the GC-MS/MS analysis, particular emphasis is placed on 9-octadecenoic acid methyl ester, which emerges as the predominant compound in NEO. This compound has been documented as an effective agent against both fungi and bacteria in prior research [47, 50, 51]. On the other hand, 9-octadecenoic acid methyl ester is also a metabolite produced by several fungal species, and certain fungi have been reported to exhibit resistance to this compound [47, 52]. Moreover, the action mechanism of 9-octadecenoic acid methyl ester was not completely elucidated, and thus it is not possible to associate the 9-octadecenoic acid methyl ester with the *P*. *citricarpa* inhibition or the cytoplasmic membrane disruption.

Several phenolic molecules were identified in NEO through GC-MS/MS analysis, including syringol, 2,6-dimethoxyphenol, phenol, and 2,4-bis-1,1-dimethylethyl, which are present in significant quantities within the NEO composition (Figure 4). Compounds containing phenol as the active principle are widely recognized as biocides [49, 53]. Syringol, 2,6-dimethoxyphenol, phenol, and 2,4-bis-1,1-dimethylethyl have been previously associated with antimicrobial properties, particularly against fungi [45, 46, 54, 55]. Despite 9-octadecenoic acid methyl ester being the predominant chemical compound in NEO, it is plausible that phenol, 2,4-bis-1,1-dimethylethyl, syringol, and 2,6-dimethoxyphenol serve as the primary agents responsible for targeting the cell membrane of *P. citricarpa*. Both phenolic molecules are known to increase membrane permeability, leading to cytoplasm leakage and subsequent cell death in microorganisms [56, 57].

The 2,6-dimethyl-1,4-benzoquinone was also identified through GC-MS/MS analysis, marking the fourth molecule previously associated with antifungal properties found within NEO [48]. Quinones, including benzoquinones, represent a class of aromatic compounds renowned for their broad-spectrum antimicrobial activity, with applications in the pharmacological field [49]. Benzoquinones are known to inhibit DNA-gyrase, a mechanism of action (MOA) that has found utility in clinical drugs [58]. DNA-gyrase, a topoisomerase present in both prokaryotes and eukaryotes, emphasizes the broad antimicrobial interest in this class of molecules. However, due to the significant abundance of syringol, 2,6-dimethoxyphenol, phenol, and 2,4-bis-1,1-dimethylethyl compared to 2,6-dimethyl-1,4-benzoquinone, coupled with their mechanism of action involving cytoplasmic membrane permeabilization, it is reasonable to consider 2,6-dimethyl-1,4-benzoquinone as a secondary inhibitory agent. If the primary mode of action was through DNA division or enzymes such as DNA-gyrase, propidium iodide would be unable to penetrate the cytoplasm and alter the color of the hyphae in fluorescence microscopy assays. Thus, the fluorescence microscope, in conjunction with GC-MS/MS analysis, confirmed the cytoplasmic membrane disruption effect induced by NEO, with phenolic molecules identified as the primary agents responsible for inhibiting *P. citricarpa*. In summary, while 2,6-dimethyl-1,4-benzoquinone and 9-octadecenoic acid methyl ester exhibit certain inhibitory activity, they do not serve as the primary agents, instead possibly potentiating the action of phenolic compounds.

**Figure 5:**
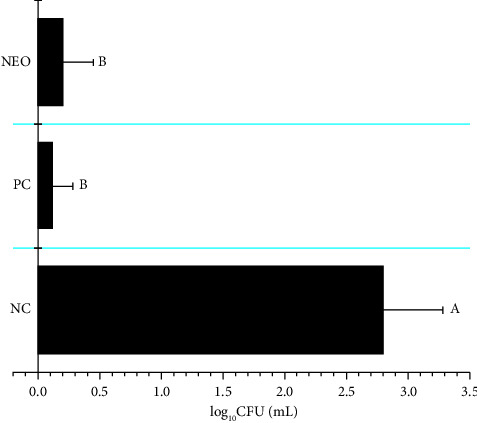
Colony forming units rescued from the surface of citrus fruits after the sanitization assays at the final time of 40 days. Bars represent the averages of rescued cells; whiskers indicate the SD of the means. Three independent experiments were performed. Data showing the same letters are not significantly different from each other based on the nonparametrical statistical analysis of Kruskal–Wallis (Dunn), with three degrees of freedom. Negative control (NC) were fruits washed using sterile deionized water with 1% of DMSO; positive control (PC) were fruits sanitized with copper oxychloride at 90 g·L^−1^ of concentration; NEO was applied at 100 *μ*L·mL^−1^ of concentration with 1% of DMSO as a vehicle. The *H* = 56.5859; *p* < 0.05 for NC to PC/NEO; the *Z* critical value was equal to 2.394 for all treatments; the *Z*-score value was 0.8957 for PC when compared with NEO, and the *Z*-score value was greater than 6.00 for NC when compared with PC or NEO.

The CBS can appear even during the postharvest phase, facilitating the spread of the disease to new areas through transportation and storage. Fungal spores are disseminated through wind, with symptoms typically appearing approximately 40 days after infection. It is crucial that all propagules of *P. citricarpa* be eradicated from fruits or any materials exported to disease-free countries, as outlined by the European Food Safety Authority [59]. Consequently, sanitization assays were conducted, and recoveries of *P. citricarpa* spores on agar plates after 40 days were performed to assess the efficacy of NEO in removing any spores or mycelium from fruit surfaces.  Figure 5 shows the microbial counts on agar plates following fruit washing with each treatment. Sanitization with NEO resulted in a significant reduction of 91.0 ± 7.35% in microorganisms (log CFU mL^−1^) compared to the negative control (NC) (Figure 5). This reduction closely paralleled that achieved with the copper-based fungicide (PC), with both treatments being statistically equivalent, thus underscoring the efficacy of NEO as an alternative for sanitizing citrus fruits in packinghouses. Notably, *P. citricarpa* was not isolated from the culture medium in any treatment except for the negative control, thus affirming the efficacy of NEO in completely eliminating any propagules of *P. citricarpa* from citrus fruit surfaces. Additionally, NEO demonstrated effectiveness in eliminating various microorganisms present on fruit surfaces, which persisted even after washing with neutral soap solution. This highlights the reduction of microorganisms on fruit surfaces and, in conjunction with the *in vitro* results (fluorescence microscopy and antifungal sensitivity assays), supports the assertion that NEO is effective in sanitizing citrus against *P. citricarpa* and other microorganisms.

It is noteworthy that, to date, no investigation has explored the use of NEO as a sanitizer for other fresh fruits. Silva et al. [39] utilized NEO against the fungi *Aspergillus flavus* and *Penicillium citrinum*, yielding favorable outcomes in protecting soybean seeds from these phytopathogens. Similarly, Khan et al. [35] demonstrated the efficacy of neem extract as an antifungal agent against mango rot phytopathogens *Lasiodiplodia theobromae* and *Neofusicoccum parvum*. These studies closely parallel our own in terms of results and application, as NEO has not previously been employed as a sanitizer against *P. citricarpa*. In addition to the novel findings presented herein, this study paves the way for further exploration of NEO's potential for sanitizing various types of fresh fruits and assessing its efficacy against other citrus phytopathogens. Many plant pathogens are categorized as quarantine organisms by the EU and other nations, and with escalating sanitization restrictions coupled with the quest for eco-friendly compounds, this study presents a viable alternative by proposing the application of essential oils such as neem for sanitizing exported fresh fruits.

## 4. Conclusion

The concentration of NEO required to achieve fungicidal action equal to copper oxychloride was found to be 100 *μ*L·mL^−1^, effectively inhibiting *P. citricarpa*. Fluorescence microscopy analysis demonstrated that NEO exerted a rapid effect, disrupting the cytoplasmic membrane of the fungus within 30 minutes of contact, thus representing the primary mechanism of action. GC-MS/MS analysis corroborated these findings, identifying phenolic compounds (syringol, 2,6-dimethoxyphenol, phenol, and 2,4-bis-1,1-dimethyl ethyl) as the major antifungal agents in NEO. The utilization of NEO as a sanitizer for citrus fruits has proven highly effective, significantly reducing the number of microorganisms present on fruit surfaces after sanitization and effectively eliminating any remaining propagules of *P. citricarpa*.

In the future, further research can be conducted on other citrus fruits such as limes, lemons, tangerines, and grapefruit. Additionally, exploration of NEO's efficacy against other fruits and microorganisms could expand the availability of organic alternative sanitizers in the global fresh produce market.

## Figures and Tables

**Table 1:** Compounds identified using GC-MS/MS spectral.

Name of compound	Neem essential oil
	RT
9-Octadecenoic acid, methyl ester	15.07
6-Octadecanoic acid	15.41
Syringol, 2,6-dimethoxyphenol	13.90
Methyl stearate	15.21
L-ascorbic acid 2,6-dihexadecanoate	14.23
Oleic acid, (2,2-dimethyl-1,3-dioxolanyl) methyl ester	5.01
Phenol, 2,4-bis-1,1-dimethylethyl	18.49
2,6-Dimethyl-1,4-benzoquinone	10.79

## Data Availability

The data that support the findings of this study are available from the corresponding author upon request.
